# Machine learning based classification of presence utilizing psychophysiological signals in immersive virtual environments

**DOI:** 10.1038/s41598-024-72376-1

**Published:** 2024-09-17

**Authors:** Shuvodeep Saha, Chelsea Dobbins, Anubha Gupta, Arindam Dey

**Affiliations:** 1 School of Electrical Engineering and Computer Science, The University of Queensland, Brisbane, QLD 4072 Australia; 2 Department of Electronics and Communications Engineering, Indraprastha Institute of Information Technology Delhi (IIIT-D), New Delhi, India

**Keywords:** Virtual reality, Presence, EEG, EDA, Machine learning, SHAP analysis, Computer science, Information technology

## Abstract

In Virtual Reality (VR), a higher level of presence positively influences the experience and engagement of a user. There are several parameters that are responsible for generating different levels of presence in VR, including but not limited to, graphical fidelity, multi-sensory stimuli, and embodiment. However, standard methods of measuring presence, including self-reported questionnaires, are biased. This research focuses on developing a robust model, via machine learning, to detect different levels of presence in VR using multimodal neurological and physiological signals, including electroencephalography and electrodermal activity. An experiment has been undertaken whereby participants (N = 22) were each exposed to three different levels of presence (high, medium, and low) in a random order in VR. Four parameters within each level, including graphics fidelity, audio cues, latency, and embodiment with haptic feedback, were systematically manipulated to differentiate the levels. A number of multi-class classifiers were evaluated within a three-class classification problem, using a One-vs-Rest approach, including Support Vector Machine, k-Nearest Neighbour, Extra Gradient Boosting, Random Forest, Logistic Regression, and Multiple Layer Perceptron. Results demonstrated that the Multiple Layer Perceptron model obtained the highest macro average accuracy of $93\pm 0.03\%$. Posthoc analysis revealed that relative band power, which is expressed as the ratio of power in a specific frequency band to the total baseline power, in both the frontal and parietal regions, including beta over theta and alpha ratio, and differential entropy were most significant in detecting different levels of presence.

## Introduction

Presence is described as the consciousness of the state of where we belong to and what we are currently feeling^1^. Presence is felt by an individual in a specific environment and state of mind^2^. Within VR, presence is the subjective feeling of “being there” in a virtual environment, which may lead to changes in consciousness^3^. In a virtual environment (VE), users can develop a unique mental model of the virtual space that differs from their understanding of the physical space. Specifically, when users are present in a VE, they perceive their own body as located within the virtual space, rather than viewing it from an external perspective. This can be understood as a specialized cognitive process that results in a distinct mental model of the VE^4^. Additionally, individual differences in cognitive engagement and the level of interactivity of the virtual environment also influence the experience of high presence and the effect of immersion^5,6^. Moderate levels of immersion can also induce a sufficient level of presence with the inclusion of an adequate amount of sensory information and cognitive belief^7^. This predominantly supports the idea that a subjective sense of presence is an effect of the objective attribute of immersion^8^. These findings have important implications for the design of virtual environments and the selection of immersive technologies in different applications^9^.

Application based research^10^ illustrates that psychomotor task performance of users in a VE can be significantly improved by training virtually in environments with high levels of presence. As such, there is a positive correlation between presence and performance. The sense of presence that VR can provide has been shown to enhance the effectiveness of training by improving motivation, engagement, and attention^11,12^. Furthermore, improved learning performance has been observed in instances where a high level of presence was reported in the VE. Maintaining VR presence is crucial in learning and training environments because it increases attention and reduces distractions, which are essential for effective learning^13^. However, a negative side effect of VR is cybersickness (CS)^14^, which can occur at any time in the VR experience but is increased with prolonged use of VR^15^ and can cause nausea, dizziness, headaches, and fatigue^16^. Nevertheless, presence appears to have a inverse effect on cybersickness^17^.

Currently, there is no established technique for measuring and detecting the real-time level of the objective experience of presence in VR using machine learning. Instead, questionnaires are commonly used to measure user’s self-reported experience of being in a virtual environment *after* they experience it. To get the most accurate and useful data possible, consideration must be paid to every questionnaire component, from design to choosing the appropriate target audience^18^. However, this method has drawbacks, including neutral responses, biased answers, and extreme choice of ratings^19^. Additionally, fixed-choice questions typically assume that the respondents have a general understanding of the subject under investigation. As a result, respondents are forced to answer questions about subjects that they may not fully understand and which they may have a different understanding based on their perceptions. Exogenous factors, such as education, culture, age, or social standing may also influence them. Since a questionnaire has no way of addressing these issues, results may be marginally biased or downright false^20^. The most significant drawback of questionnaires is the inability to self-report in real time, as they are often deployed after a scenario, which requires participants to rely on their memories when providing their responses^21^. Moreover, this can be a challenge for researchers trying to collect accurate data about users’ experiences or attitudes towards a particular task or situation^22^. To overcome this issue in VR, questionnaires could be deployed in the environment at regular time intervals. However, this could break the feeling of presence, and negate the whole idea of maintaining and quantifying presence^23^.

To overcome these limitations, psychophysiological signals recorded from the brain and body provide an objective measure that can be used to monitor a user’s real-time autonomic response during a task against a stimuli. Such signals include neurological signals, such as Electroencephalography (EEG), and physiological signals, including Electrodermal Activity (EDA) or Skin Conductance (SC), Photoplethysmography (PPG), Electrocardiography (ECG), Heart Rate (HR), Skin Temperature (ST), Electromyogram (EMG), and pupil dilation^24,25^. The effect of presence can, therefore, be objectively measured by obtaining both the users’ feedback (via questionnaires) and psychophysiological signals. This work advances the current state-of-the-art by detecting presence using psychophysiological signals and machine learning to provide an objective measure of presence.

The long-term goal of this research is to develop a methodology to quantify presence in real-time. Such a system will be beneficial in enhancing the user experience of VR and, hence, improve the effectiveness and quality of VR-based applications and interventions. To advance this idea and to address the gap in the literature, the current study makes the following salient contributions:

1. Development of a multimodal data processing pipeline for both EEG and EDA signals that processes multiple streams of neurological and physiological data to detect presence.
2. Evaluation of several machine learning models to detect three different levels of presence (high, medium, and low) in VR. In this case, presence was manipulated using parameters such as graphics fidelity, audio cues, latency, and embodiment with haptic feedback.
3. Identification of important features that can discriminate between different levels of presence, including relative band power and Higuchi Fractal Dimension (HFD) based features from the frontal region and entropy of phasic skin response in higher presence

The findings of this research have significant implications for enhancing the effectiveness and usability of VR-based interventions and applications across various domains, including healthcare, education, and entertainment. This is the first study to build a machine learning model to detect different levels of presence and identify relevant psychophysiological features that can be directly used in future studies to construct better real-time user-experience-centric VR applications.

## Related work

Different aspects of a VR environment can affect the feeling of presence and immersion, including visual quality, embodiment, sensory stimulation. Furthermore, immersion and place illusion (two prevalent high-level concepts), are distinctive objective and subjective features that can also affect different aspects of presence in VR^26,27^. The following section provides an overview of related work in this area, as well as the use of psychophysiological signals in detecting presence.

### Visual quality

Place illusion is the perception of being in a virtual location while one is actually not there. Geometric realism is a key component of VR, as it provides insight into how realistic virtual items are compared to real-world objects. Another important parameter is illumination realism, which controls lighting fidelity^28^. Different aspects of visual stimuli are critical in creating a realistic VR experience for the user. These include the size and resolution of the screen, the use of stereo and head-tracking in a head-mounted display (HMD), and the use of real-time motion capture. For instance, a study with 33 participants^29^ investigated levels of presence by dividing participants into two groups that each experienced a different level of graphical rendering. One group experienced ray tracing, which was full recursive tracing that had more detailed visual quality. The other group experienced ray casting, which was pixel-based illumination. Participants from the ray tracing group reported increased presence than the ray casting group.

Additionally, the realness of the virtual world can be evaluated based on the level of detail in the graphics, such as the number of triangles used in 3D models and the resolution of textures applied to surfaces. By optimizing these visual stimuli, VR can provide a more immersive and realistic experience and increase the level of presence that is felt^30^. A pioneering study by Palmisano et al.^31^ demonstrated a direct correlation between display lag and perceived scene instability. The study was undertaken with 30 participants who undertook a Directional Vertical Pursuit task that measured the level of scene instability that participants experienced while using a HMD with varying degrees of display lag. Results illustrated that as display lag increased, participants reported increased levels of scene instability. This suggests that reducing display lag can improve the overall user experience and increase immersion in the virtual environment.

### Embodiment

A crucial component of presence that underpins its multidimensional character is perceived realism^32^. One of the key factors in perceived realism is embodiment, which can be divided into three groups: *self-location*, *sense of agency*, and *body ownership*. Sense of agency relates to the correlation between the actions of the users real body part to those of the virtual character, while self-location provides a sense of similarity between the virtual body’s position and the users actual position. Body ownership relates to the perception of one’s own body inside the virtual character. These subcategories are connected and all contribute to improve embodiment^33^.

Lankoski et al.^34^ hypothesized that when players were embodied in a first-person view character in a gamified world, even in desktop VR, they experienced improved sensory-motor coordination. This gave people the impression that they were physically there in the game. While first-person and third-person perspectives did not significantly differ, there was a significantly high correlation between embodiment and presence. In an HMD-based study^35^, researchers used three different conditions—embodied, included, and observed—to investigate the impact of embodiment on presence. The embodied condition involved the participant controlling a virtual character, the included condition involved the participant being included in the virtual environment without controlling a character, and the observed condition involved the participant watching someone else control a virtual character. The results demonstrated that the embodied condition led to the highest level of presence, compared to the included and observed conditions. This suggests that embodiment plays an important role in creating a sense of presence in virtual environments. Additionally, recent research on the relevance of individual variables suggests that in VR, control and first-person perspective, over an avatar, are more crucial for embodiment than how the avatar looks^36^.

### Sensory stimulation

Supporting additional sensory channels, such as tactile, olfactory, and taste feedback, in accordance with the context of the specific VR experience, can also heighten the impression of realism^37,38^. For instance, a study by Kern et al.^39^ was undertaken in a virtual “walk-in-the-park” environment to highlight the role of auditory stimulation in augmenting the perceived sense of presence. The study consisted of two separate experiments. The first experiment had footstep sounds and ambient soundscapes, whilst the second had the same thing, along with a high-quality reproduction of step sounds that were synchronized with the participants’ actual footsteps. Results demonstrated the sound of self-triggered footsteps in the second experiment had a more realistic effect compared to the first, where only soundscapes were used. This study concluded that the addition of a variety of sounds (e.g. ambient nature soundscapes and movement-triggered step sounds) led participants to have a stronger impression of the real world.

Moreover, along with other sensory feedback, the most important is haptic feedback, which provides a greater sense of immersion^40^. Many studies have found that including haptic feedback significantly improves task performance^41,42^. However, results were inconclusive in relation to presence. For instance, research conducted by^15^ found that haptic feedback in a virtual environment improved task performance, but did not have a significant impact on the sense of presence. The authors suggest that haptic feedback may not be as important for presence as visual and auditory cues. However, other research has found that haptic feedback can sometimes interfere with presence in virtual environments, particularly if the feedback is not synchronized with other sensory cues^43^. Hence, it is necessary to provide this type of feedback simultaneously with other sensory cues.

### Neurological and physiological signals

EEG is the most widely used neurological signal for investigation into presence^44–49^. For instance, Baumgartner et al.^47^, investigated the effect that different levels of immersive effects had on presence and EEG signals during a simulated roller coaster ride. Localization of EEG activity was observed over the parietal lobe when event-related cues were presented to the participants. This demonstrated that parietal lobe activation was more prominent in engagement-inducing scenarios in VR. In a similar study^48^, participants experienced a first-person roller coaster ride as a virtual video game whilst EEG data was collected. Self-report data was also gathered via the Presence Questionnaire (PQ), Slater, Usoh, and Steed (SUS) Questionnaire^50^, and a short version of the Multidimensional Personality Questionnaire^51^. This study was based on task-dependent Event Related Potential (ERP), where they investigated the mean amplitude value of ERP for the first negative peak N1 (100–200 ms), mismatch negativity (MMN) (150–200 ms), early slow-wave (SW1) (400–650), and late slow waves (SW2) (650–900 ms) components. Slow waves descended in the negative region with low presence, whereas no such distinguishable difference between low and high was observed in N1 and MMN. The amplitudes of the SW1 and SW2 ERP components were significantly correlated with all of the questions.

Physiological signals have also been used to measure sympathetic and parasympathetic responses in VR environments^46,52,53^. Sympathetic activity refers to the sudden activation of the sensory response against a specific situation, known as the fight or flight response, while parasympathetic activity denotes returning to a relaxed state^54^. For instance, Meehan et al.^52^ compared participants’ physiological reactions when in a stressful virtual height vs a non-threatening virtual room environment. Physiological data, including HR, SC, and ST, as well as the University College of London Questionnaire, were collected. Results illustrated that presence was strongly correlated with a change in HR and, to a lesser extent, SC. There were no changes in ST. Similarly, Busscher et al.^53^ studied heart rate using a phobic stressor of a virtual flight scenario. Participants were distributed amongst two groups (phobic and non-phobic) that each experienced three conditions: neutral virtual world, virtual flight, and recovery. Self-report data was also collected, including The Igroup Presence Questionnaire (IPQ)^55^. Results demonstrated that HR was highest for both groups during the neutral environment compared to the other conditions, whilst the IPQ scores of the non-phobic group were significantly higher.

### Classification of presence in VR

Machine learning approaches have sparingly been used to detect and classify presence^56,57^. For instance, Savalle et al.^56^ explored the use of EEG to monitor and classify users’ levels of presence in VR based on reactions to oddball stimuli. Participants experienced VR conditions with and without disruptions while EEG data were recorded. Riemannian geometry was applied to classify the reaction to the stimuli. The classifiers utilised 5-fold cross-validation to achieve accuracies between 67 and 80%, depending on the number of electrodes used and the epoch duration.Th best performance was observed in the mid-frontal and pre-frontal region of brain. Additionally, Ochs et al.^56,57^ utilised a random forest model to predict presence and co-presence in a virtual reality environment using verbal and non-verbal cues in a virtual clinical environment. Deep Learning^58^ can also be used in this space. However, currently, these methods have been applied largely for the detection of stress using convolution neural networks^59^, emotion^60^ and cybersickness^61^.

The majority of studies classify either self-reported levels of presence or neurological responses in VR environments. As such, there is a gap in the literature in utilising physiological signals to detect presence experienced throughout the VR experience, which can be used to improve the overall user experience. Further research is required to develop a detection method that can measure presence using psychophysiological responses in real-time. As a first step in this direction, this paper identifies the optimal feature set and best machine learning models to classify levels of presence.

## Materials and methods

### Participants

The study consisted of 22 participants (8 females and 14 males), whose age ranged between 20 and 50 years (mean = 28.77, SD = 7.09). Participants had no history of cardiovascular illness (e.g., heart disease, arrhythmia) and/or seizures, did not take any medications that affected the heart, or were not pregnant at the time of the study. The study was approved by The University of Queensland Research Ethics Committee. The study was conducted in accordance with Australia’s National Statement on Ethical Conduct in Human Research and the principles expressed in the Declaration of Helsinki.

### Design of virtual environments

**Fig. 1:**
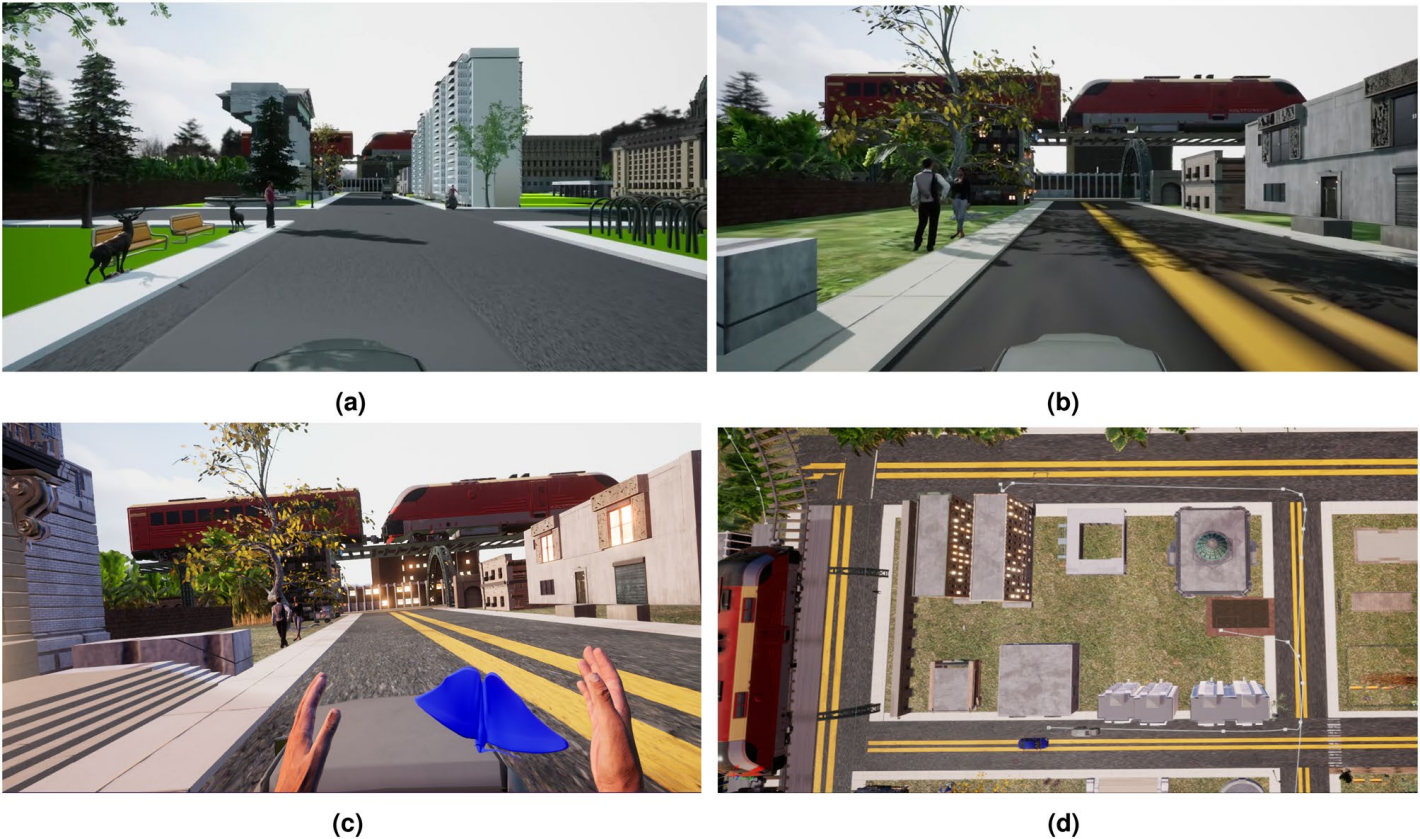
Developed virtual environments for the three different presence levels: (**a**) Low presence scenario with lower visual quality, no embodiment, no audio cues and induced latency, (**b**) Medium presence with medium visual quality, intermediate latency, no body control and audio cues, (**c**) High presence scenario with high visual quality, embodiment and haptic feedback, no latency and detailed audio cues, (**d**) Top view of the designed environment. Figures were created by taking screenshots of the environments that were created using Unreal Engine 5.0^62^.

A scenario of a town has been created using the Unreal Engine platform (UE 5.0)^62^ and available free assets from the Unreal Engine marketplace^63^. Three different versions of this environment were developed to create low (Fig. 1a), medium (Fig. 1b), and high (Fig. 1c) presence scenarios. As higher cognitive load can reduce the level of presence^64^, the environments were designed to minimise cognitive load. This was achieved by not requiring participants to engage in many tasks and thus interaction was kept to a minimum. The participant’s character travelled along in an automatically driven truck, along a pre-determined path, where the participant could move their head freely and observe everything around them, but they could not move from their position. The town environment was designed such that there were buildings of different sizes, a children’s park, a fountain, trees, an overhead train track and static train compartments. Fig. 1d provides an overhead view of the designed environment.

Moreover, there were five animated and one static human avatars placed in different places throughout the scene. Several butterflies were also placed in specific spots in the environment that were within close proximity of the participant’s character. Variables in these scenarios that were manipulated were sourced from the literature^31,35,39,65^ and are described below.

The fidelity of the graphics was manipulated using texture and the details of the assets and objects in the environment^65^. In the low presence scenario, solid color was used for the ground and road without any texture or added foliage. Additionally, building texture and lighting were also not provided. Moreover, human avatars in the environment did not move. In the medium presence scenario, realistic ground and roads were designed, although no fine foliage was included in this scenario. Building texture (without lighting) and animations of human avatars moving were also included. In the high-presence scenario, roughness and decals were added to the landscape materials. Foliage was used as ground cover, with small and large plants of different shapes. Buildings contained more realistic color, texture, and lighting to provide a more detailed visual experience. However, the human avatars were the same as in the medium presence scenario.

Audio and latency were also manipulated, as high latency can negatively affect immersion by creating a visual mismatch between the participant’s action and what they are perceiving^31^. In particular, 100 ms, 50 ms, and 0 ms time delays were induced in the low, medium, and high scenarios, respectively. The low presence scenario did not include audio, whilst the medium scenario included environmental sounds. The high scenario included additional sounds of the vehicles, along with the environmental sounds^39^.

Embodiment, including haptic feedback, was also adjusted^35^ In low presence, the character of the participant had no visible figure. However, in medium presence, the character was embodied in an avatar that could not be moved or controlled by the participant. In the high presence scenario, the character was embodied in a human avatar, from a first-person perspective, and the participants were given control to move their virtual hands using motion controllers. In addition, haptic feedback was introduced in the high presence scenario when the participant tried to catch butterflies located within the environment. Eight butterflies were positioned in the environment at certain distances, which appeared at around specific time instances. In the low and medium presence environments, they could not interact with the butterflies due to the absence of embodiment. Trigger buttons of the controller were used to perform this action.

### Hypotheses

It was expected that the different levels that were designed to induce high, medium and low presence, would sufficiently affect the level of presence that participants felt. Additionally, it was expected that this shift in presence could be detected and classified using psychophysiological signals and machine learning. As such, the study has been informed by the following hypotheses:

1. Variation of graphics fidelity, latency, audio cues, and embodiment can successfully alter the level of presence in the virtual environment.
2. Self-reported presence scores corroborate that the levels were inducing the anticipated level of presence that was expected.
3. Features derived from psychophysiological (EEG and EDA) signals can detect and classify levels of presence.

### Study design and procedure

The study was conducted in a within-subjects design, where all participants experienced all the VR environments. Before beginning the study, participants were provided with a description of the study, including a description of the whole experiment set up, including time duration of the individual environments, resting time and number of repetitions. Once written informed consent was obtained from all the participants for their voluntary participation, study commenced. All participants were remunerated with a gift card of $10 AUD once they had completed the study.

**Fig. 2:**
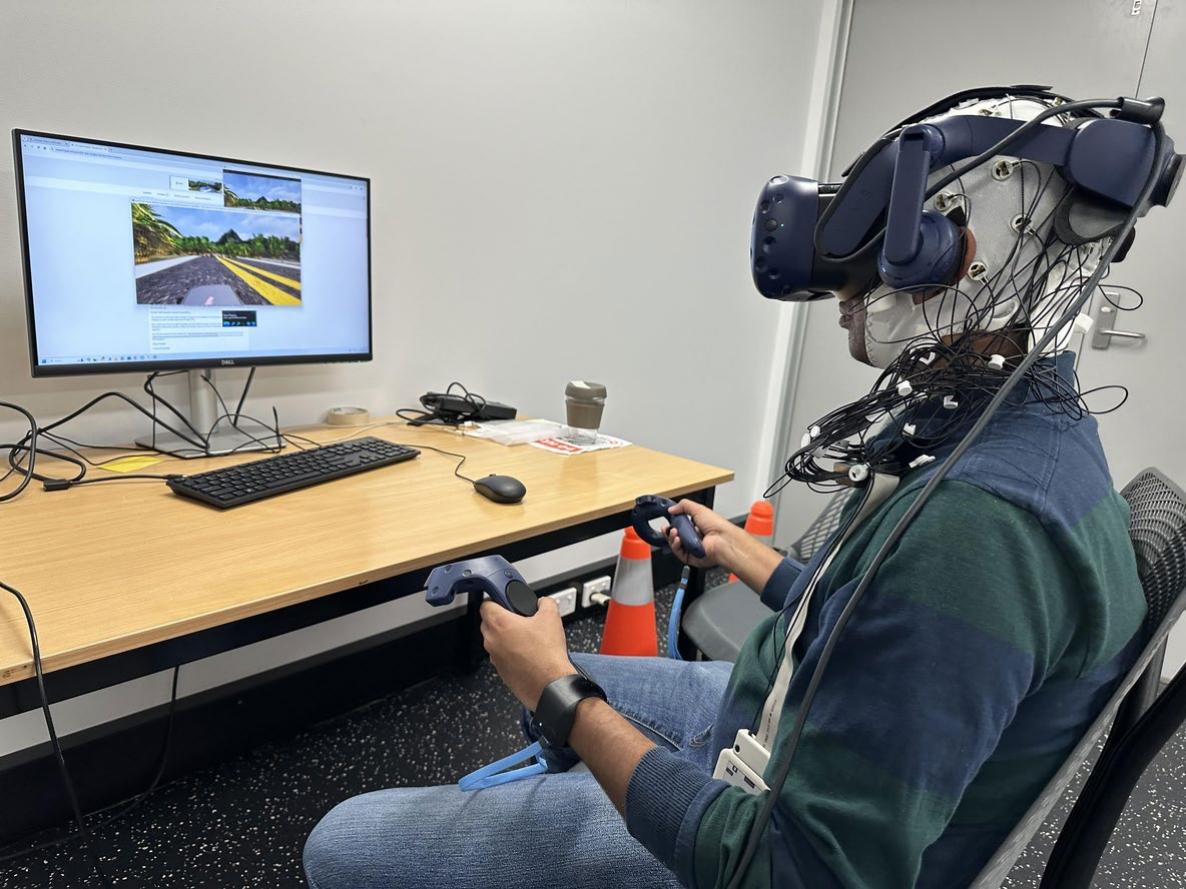
Setup of the study that included the HTC VIVE Pro VR headset, BrainProducts LiveAmp EEG system and Empatica E4 wristband.

An HTC VIVE Pro was used to deploy the VR experiences. EEG data was recorded using a 32-channel BrainProducts LiveAmp system at a sampling rate of 250 Hz, whilst EDA was obtained using an Empatica E4 wristband at a sampling rate of 4Hz (see Fig. 2) . The standard montage of Actichamp LiveAmp was used, taking FCz as reference and FPz as ground. Data was recorded from 20 channels, including two from the Prefrontal region (FP1, FP2), four from the Frontal area (F3, F4, F7, F8), two Fronto-temporal (FT9, FT10), four Central-frontal (FC1, FC2, FC5, FC6), four from the Parietal region (P3, P4, P7, P8), two from the Occipital area (O1, O2) and one each from the mid-frontal (FZ) and mid-parietal (PZ) regions. The whole experiment lasted approximately one hour, including 30-40 minutes to set up the system.

**Fig. 3:**
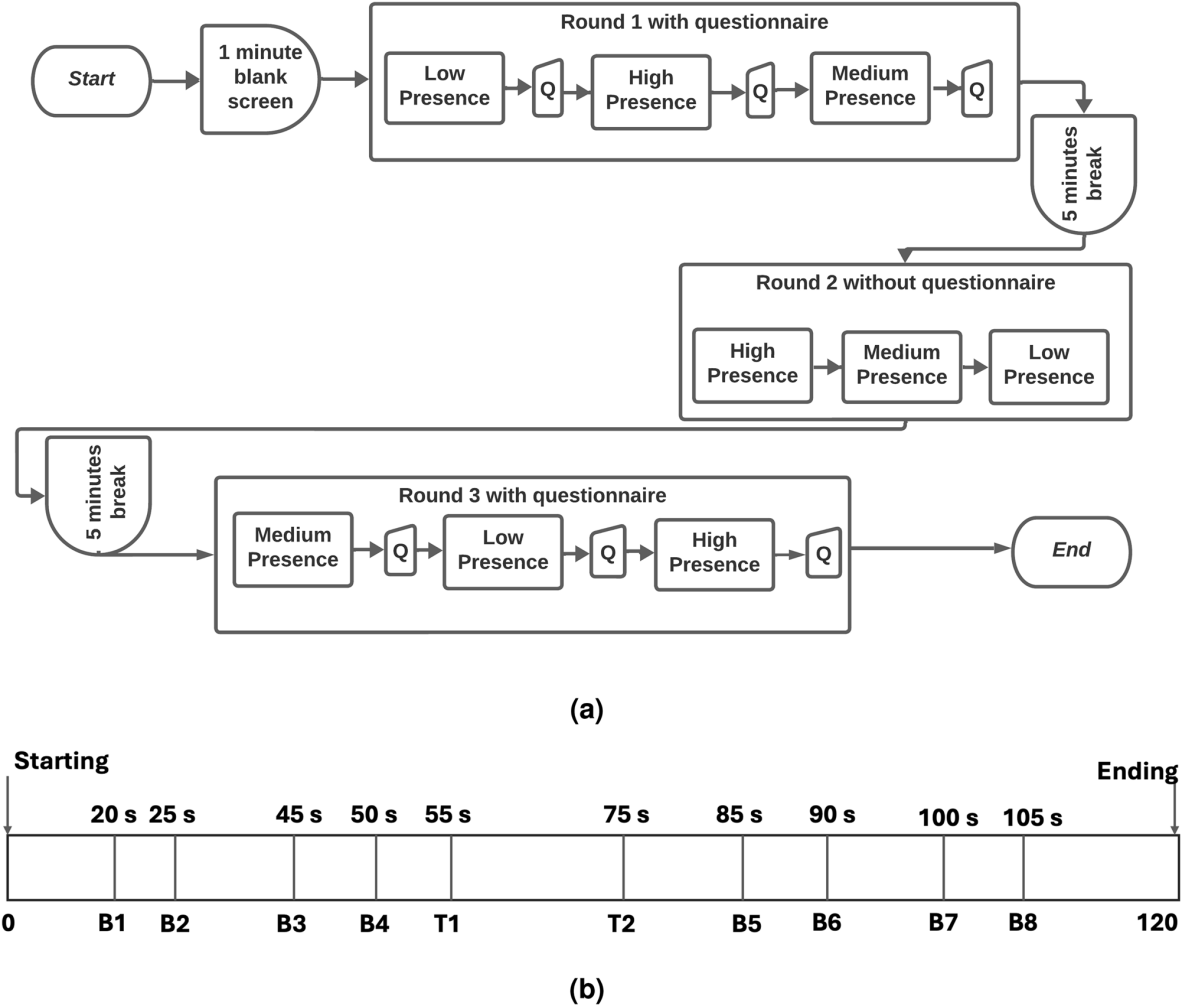
Experimental design (**a**) Three rounds with breaks where ’Q’ stands for Questionnaire and (**b**) Timeline for a single presence scenario, where ’B’ stands for Butterfly, ’T’ stands for Turning of road, and ’s’ stands for seconds.

The entire data collection procedure took place over three rounds (see Fig. 3a). Participants were requested to restrict their body movement as much as possible, except head movements and required hand movements. During each round, participants were presented with the three different presence scenarios. As the task consisted of three rounds, the order of the scenarios was counterbalanced in each round to minimize order effects. As such, there were six possible sequences for the three scenario conditions, i.e. HML, HLM, MHL, MLH, LHM, LMH, where H, M, and L denote high, medium and low presence scenarios, respectively. For each participant, one sequence was randomly chosen for the first round, and then Latin Square counterbalancing was applied to the remaining two rounds. For example, Fig. 3a shows one task setup, where the first round was randomly chosen to be LHM. Using the Latin Square method, the remaining two rounds were selected to be HML and MLH. After attaching the sensors and headset, but prior to experiencing the virtual environments, a one minute baseline was recorded, where participants were asked to sit still and stay calm. Although their headset was on, only a black screen was displayed. Fig. 3b illustrates a timeline for a single presence scenario, which depicts distinct complexities that occurred in the environment. For instance, eight butterflies were placed in the environment, which participants passed by at certain time points. The predefined track that they travelled down also had two turning points in the road that was experienced by the participants.

Along with the sensors, the IPQ^55^, was used to collect self-reports about their feelings of presence. This questionnaire comprised a total of thirteen questions. The questionnaire was completed after each scenario during the first and third rounds. The questionnaire was not deployed during the second round in order to minimise the effect of fatigue and deviation from the target behavior due to adaption^66^. After each round, participants rested for five minutes, at which point they could remove the VR headset. However, the sensors were kept attached. All the signals and connections were checked thoroughly before each round of data recording. Participants were exposed to the VR environment for a total of 18 minutes. This comprised of six minutes per round, whereby they were exposed to each level for two minutes within each round.

### Experimental measures and data pre-processing

The IPQ questionnaire was used to measure presence experienced by the participants during the study. The IPQ consisted of a 7-point Likert scale, where 6 represented the highest level of presence, and 0 represented the lowest level of presence felt by the participants.

Raw EEG and EDA data were processed using MATLAB R2021a. Firstly, a 50 Hz notch filter was applied to remove power line interference in the EEG signal. As EEG is a highly non-stationary signal with a wide frequency range, whereby the mean and variance of the signal do not substantially vary over time, it was necessary to filter the signal in a specific frequency bracket. A Butterworth high pass filter of order 6, with a pass band frequency of 1 Hz, was used to discard very low-frequency components. A low pass filter was then applied with a cut-off frequency of 30 Hz. The whole dataset was then normalized for zero mean and unit standard deviation. Furthermore, wavelet-enhanced Independent Component Analysis (wICA)^67^ was utilised to remove eye blinks and other movement artefacts that were present in the signal. This algorithm is based on FastICA^68^ that decomposes a multi-channel EEG signal into multiple independent components, where the components with repetitive patterns are recognised as eye blink artefacts. This algorithm was chosen over traditional manual inspection because it does not distort neural activity, thus preserving both spectral and phase characteristics^69^. Additionally, the algorithm uses a wavelet threshold to automatically detect low-amplitude movement noise. Reconstruction of the multi-channel signal, using artefact-only independent component analysis (ICA) components, yields EEG signals that consist only of noise. These noisy reconstructed signals were then subtracted from the original signal to produce noise-free signals. The baseline period was used to derive the relative band power for each participant. However, linear or log-power normalization was not undertaken because there was no event related instances within the study, which needed to be normalized against the non-event baseline. In the case of event related studies, background neural activity might be present in the desired event activity and influence the signal power^70^. However, in this study, the overall response is considered as the desired response towards environment.

The raw EDA data was processed using a low pass Chebyshev filter, with a 1 Hz cut-off, to remove high-frequency data, which is generally considered as high-frequency motion artefacts^71^. The data was then normalised using a zero mean and unit standard deviation. EDA is composed of two components - phasic Skin Conductance Response (SCR) that reflects the response against sudden stimulation, and tonic Skin Conductance Level (SCL), which indicates slow changes over time. Both components were extracted from the EDA signal using the cvxEDA (Convex Optimization Approach to Electrodermal Activity) algorithm^72^. This method aims to separate the tonic and phasic components of the EDA signal, which are respectively associated with baseline activity and rapid changes in skin conductance level that reflects sympathetic nervous system activity. The approach is based on convex optimization, which enables the estimation of the tonic and phasic components of the signal while imposing constraints that reflect the physiological properties of EDA^72^.

#### Feature extraction

After the data was pre-processed, 17 EEG channels were selected for feature extraction, which is in line with the previous works^73,74^. These included the frontal channels (F3, F4, F7, F8), Pre-frontal channels (FP1, FP2), Fronto-temporal channels (FT9, FT10), mid-frontal at FZ, Central-frontal (FC2, FC5, FC6), Parietal (P3, P4, P7, P8), and mid-parietal at PZ. The frontal and parietal regions have been chosen because the parietal lobe is responsible for perception, processing of information and spatial awareness^75^, whereas the frontal lobe accounts for overall cognitive and emotional operations^76^. The Occipital region (O1, O2) was excluded from the analysis as the focus of the study was focused towards participants perception of the environment, which is more applicable to the frontal and parietal lobes^8^.

The EEG data was first decomposed into theta (4–8 Hz), alpha (9–12 Hz) and beta (13–25 Hz) bands using Morlet wavelet. For each band, root mean square band power was calculated for each presence scenario (low, medium, and high) and the baseline (resting state without VR). Each presence level recorded approximately 100 s of data, which was divided into 29 epochs of 5-s windows, with 30% overlap. This overlap was chosen as it reduces edge effects that occur when the windows are fully separated^77,78^. The decision to include a 30% overlap was informed by previous studies that illustrated that this overlap was sufficient to preserve more temporal data and reduce edge induced errors^79^. Features were then extracted from the EEG and SCR/SCL data per window.

For the EEG data, a ratio of power in each epoch to the baseline power was individually calculated for all the presence levels in the alpha ($\alpha$), beta ($\beta$), and theta ($\theta$) bands for every epoch of every channel. These power ratio features for different frequency bands are henceforth called the relative power features of $\alpha$, $\beta$, and $\theta$ bands. The features included $\frac{frontal_\theta }{parietal_\alpha }$^80^ calculated as the ratio of frontal ( Fz, F3, F7, F4, F8) to parietal (Pz, P3, P7, P4, and P8), $\frac{\beta }{(\theta +\alpha )}$^81^ from channels Fz, F7, F8, P7, P8, Pz, and $\frac{frontal_\theta }{frontal_\beta }$^82^ from channels FP1, Fz, F3, F7, F4, F8, FP2. These features are also known for their determining factors of mental load and engagement^80–82^. Additionally, differential entropy (DE), which indicates the level of emotional engagement^83^, was calculated for all three frequency bands. DE is defined as:

1 $$\begin{aligned} DE = \frac{1}{2} \log (2\pi \sigma ^2), \end{aligned}$$

where $\sigma ^2$ is the variance of the time-domain signal. HFD^84^ has also been used to investigate the processing of neural activity generated towards their response to different complexities of the environments corresponding to three different presence scenario designs.

Statistical features were extracted from the SCR/SCL signals, which included mean, median, standard deviation, kurtosis, and Shannon entropy. The normalized power spectral density (PSD) of both components were also used as features.

### Machine learning models

A number of classifiers were designed to distinguish between the three levels of presence. These included Support Vector Machine (SVM), k-Nearest Neighbour (kNN), Extra Gradient Boosting (XGBoost), Random Forest (RF), Logistic Regression (LR), and Multiple Layer Perceptron (MLP). The study was designed as a three-class classification problem, using a One-vs-Rest approach, to test the model’s abilities to classify the three different levels of presence (classes). Each of the models were validated using the leave-one-person-out cross-validation (LOOCV) method, whereby one participant was randomly selected for testing, whilst the data of the rest of the participants were used as the training set. This process was repeated until the data of all participants were used as the test set (i.e., 22 folds) once.

Hyperparameters were tuned separately for each model using the training folds. The hyperparameters obtained for the best model were: leaf size of 1 and 13 neighbours for KNN; logistic activation function, 1 hidden layer with 25 neurons, and learning rate of 0.0001 for MLP; $\alpha$ of 1, maximum depth of 7, $\eta$ of 0.01, and the number of estimators equal to 100 for XGBoost; maximum features equal to the square root of total number of input features, minimum no. of samples at a leaf of 92 samples (5% of total training samples at epoch level), and 20 estimators for Random Forest; C of 100, gamma of 0.001, and sigmoid kernel for SVM and; no penalty and Newton-cg solver for Logistic Regression.

The data was labelled according to the environment that the participant was experiencing (i.e., data recorded during the low presence experience was labelled as “low” and so on). As such, the classification problem contained three classes: low, medium, and high. Additional analysis of the IPQ data has been undertaken to validate the labels to determine whether participants’ feelings of presence corresponded to the environment they were in, which further supports the labelling approach (see Results section).

#### Feature selection

The entire feature set included 181 features, which contained 171 EEG features and 10 SCR/SCL features. The EEG feature set comprised of nine features for each of the 17 channels and the relative power features, which were extracted from the specific channels or channel combinations detailed above (17 channels x (3 bands $\theta$, $\alpha$, and $\beta$) x 3 features (relative band power, DE, and HFD) = 153 features + five features of $\frac{frontal_\theta }{parietal_\alpha }$ + seven features of $\frac{frontal_\theta }{frontal_\beta }$ + six features of $\frac{\beta }{(\theta +\alpha )}$ = 171 features). Due to the high dimensionality of the feature set, evolutionary-based feature selection was undertaken to reduce the feature space. Genetic algorithms (GA), first introduced by John Holland^85^, are derived from Charles Darwin’s theory of natural selection to return an optimized solution for finding the best set of features for a specific model. GA was used to extract the most important features. This algorithm follows the natural selection process, where the optimal features from each random subset of the whole feature set were obtained as the best fit that were used in the classifiers.

#### Performance metrics

The performance of the models were validated using the following performance metrics^86^.

- *Accuracy:* Accuracy is an overall measure of performance that was calculated by dividing the proportion of accurate predictions by the total number of observations in the dataset.
- *AUC (Area Under the Curve):* AUC measures the quality of a model’s predictions. It represents the area under the Receiver Operating Characteristic (ROC) curve, which plots the True Positive Rate (TPR) against the False Positive Rate (FPR) at different classification thresholds, where TPR is the correctly predicted positive class and FPR is the falsely predicted positive class. AUC ranges from 0.0 to 1.0, with a higher value indicating better classification performance.
- **F1 score: ** F1 score combines precision and recall into a single score. It is the harmonic mean of the proportion of genuine positives among all projected positives, as measured by precision, and the proportion of true positives among the observed positives, as measured by recall. A higher value denotes better classification performance. F1 score ranges from 0.0 to 1.0.

Post-hoc analysis has also been undertaken on the best performing model using SHapley Additive exPlanations (SHAP), to explore the contributions that each feature has made to the results. SHAP is a popular framework for interpreting the output of machine learning models and is based on the concept of Shapley values from cooperative game theory to provide a unified approach to explain the predictions of different types of models^87^. In the context of machine learning post-hoc analysis, Shapley values can be used to explain the contribution of each feature *i* to the model’s prediction *y*. The characteristic function *v* is defined as the model’s prediction for a given input, and *N* is the set of all features. The Shapley value of feature *i* has been calculated as:

2 $$\begin{aligned} \phi _i(v) = \frac{1}{|D|!}\sum _{S \subseteq D \backslash {i}} \sum _{\sigma \in \Gamma (S \cup {i},D)}(v_\sigma (x_S \cup {i}) - v_\sigma (x_S)), \end{aligned}$$

where $\phi _i(v)$ is the Shapley value of feature *i*, *D* is the set of all features, *S* is a subset of features, $x_S$ is the input with only features in *S*, and $v_\sigma (x_S \cup {i})$ is the model’s prediction for the input with features in *S* and feature *i* set to its original value under permutation $\sigma$. This formula calculates the Shapley values for each feature in a given input, and the values can be used to explain the model’s prediction for that input. SHAP assigns a score to each feature in a given input, which represents the contribution of that feature to the model’s output. This score can assist in understanding how the model arrived at a particular prediction and can identify which features are the most important in driving the model’s decision-making process for each class.

## Results

The data analysis approach first examined the subjective self-reports to compare statistical differences between presence levels, whilst the EEG and SCR/SCL features were tested first for statistical significance. Finally, the machine learning models were evaluated for their accuracy in detecting presence using the neurological (EEG) and physiological (SCR/SCL) features. The best model has then been selected for post-hoc analysis.

### Self-report questionnaire results

**Fig. 4:**
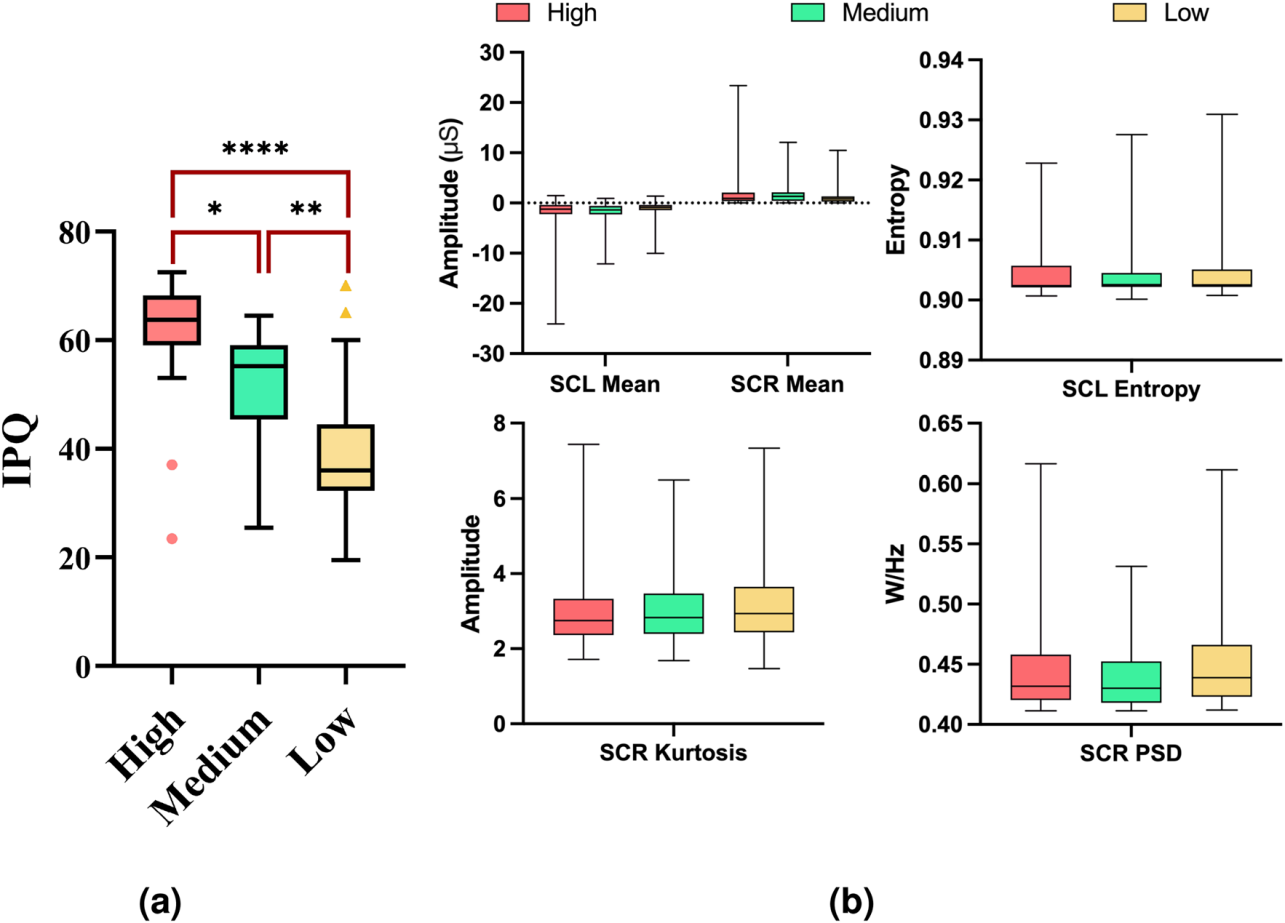
Self-report IPQ questionnaire and SCR/SCL signal analysis: (**a**) Results of a comparison among the IPQ results of the different levels, *$p<$0.05, **$p<$0.001 ****$p<$0.00001. (**b**) Comparison of the three levels of presence against the most significant SCR/SCL features.

The IPQ questionnaire data has been evaluated for statistical significance across the three levels using the Friedman test, as the Shapiro-Wilk normality test determined that the distributions were not normally distributed. Fig. 4a demonstrates that the IPQ scores were significantly different, with results of $\chi ^2$(2,N=22)= 31.18, $p < 0.001$, was observed among the three levels of high (*M*= 61.27, *SD*= 11.48), medium (*M*= 52.11, *SD*= 10.27) and low (*M*= 49.68, *SD*= 12.37). Moreover, post-hoc analysis was undertaken using Dunn-Bonferroni correction, which illustrated that the low presence level was significantly lower than the high presence level ($p<$0.001), which was reported in the questionnaire. There was also a significant difference between high and medium presence (*p*=0.03), where the medium presence score was less than the high presence score in the questionnaire. Moreover, a significant difference (*p*=0.007) was observed between the low and medium presence levels. These results validate the labelling approach used in the machine learning analysis, as participants felt elevated levels of presence in the “high presence” level, decreased presence in the “low presence” level and a moderate amount in the “medium presence” level.

### Physiological signal results

The SCR/SCL features were individually evaluated for statistical significance across the three levels using a repeated measures Analysis of Variance (ANOVA) approach. Results illustrated that the most significant features were tonic SCL Mean *F*(2,954)= 7.588, $p < 0.001$, SCL Entropy *F*(2954)= 6.39, $p = 0.001$, SCR Mean *F*(2,954)= 7.428, $p < 0.001$, SCR Kurtosis $F(2954)= 7.617$, $p < 0.001$ and SCR PSD *F*(2,954)= 11.49, $p < 0.001$. Tukey post-hoc analysis revealed that the feature set was not able to significantly differentiate between medium and low levels of presence. Fig. 4b illustrates a comparison of these top five SCR/SCL features that have been described above.

### Neurological signal results

**Fig. 5:**
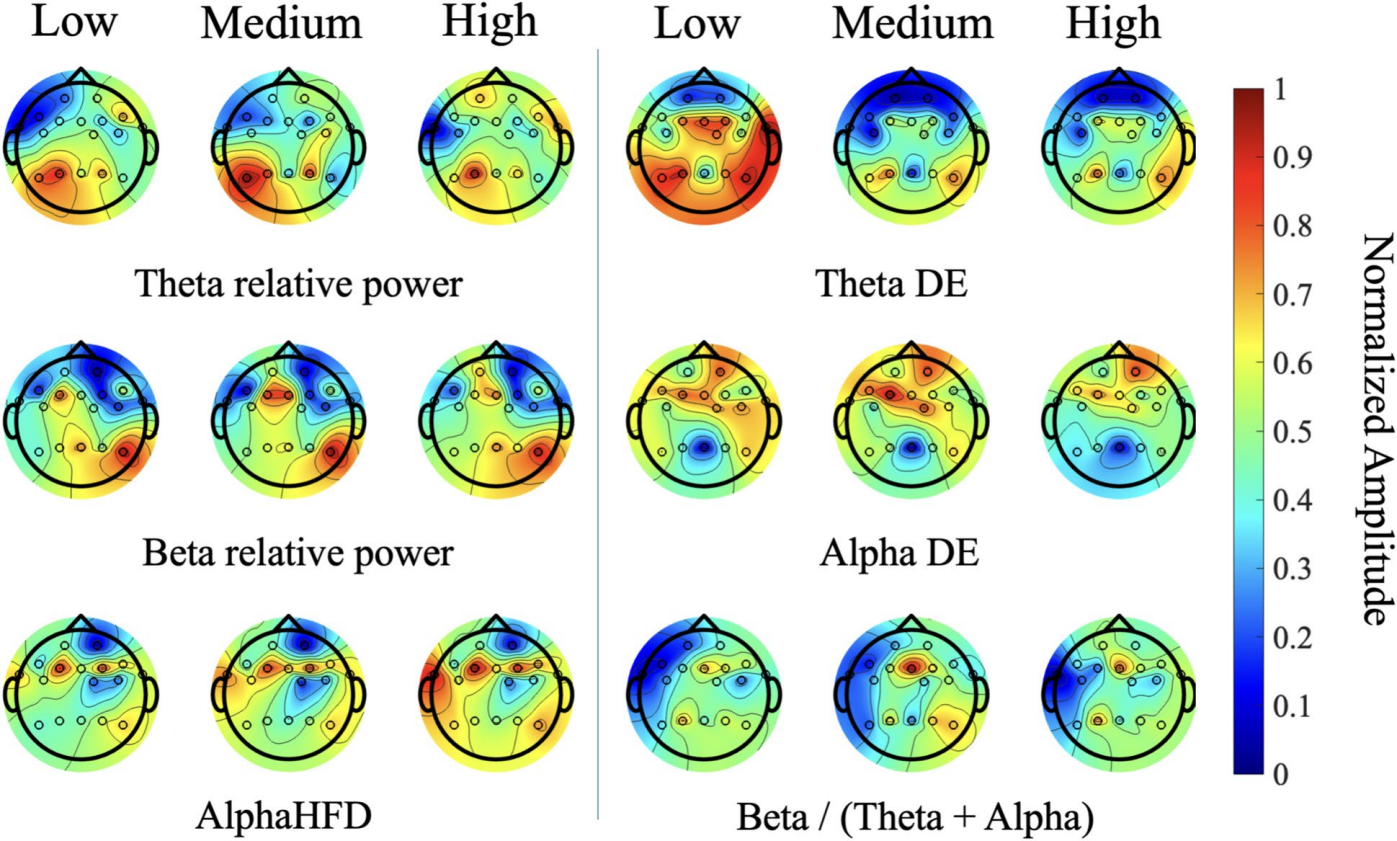
Topographical plots of the highest-ranked EEG features across the three presence levels.

The EEG features were individually evaluated for statistical significance using a repeated measures ANOVA. The results demonstrated 78 features produced highly significant results, whereby $p < 0.05$. The topological maps in Fig. 5 illustrate a sample of the highest-ranked features to visually depict differences in features across the various presence levels. The results in this figure have been normalized for each feature set. As such, the colorbar represents this normalized scale. The results illustrate that during the high level, relative power of the $\theta$ and $\beta$ bands increased in the pre-frontal and mid-parietal regions, respectively. Significant changes in fractal dimension were also observed in the fronto-temporal and tempo-parietal areas, with larger values being exhibited in the $\alpha$ band. In contrast, DE was higher during the low level and decreased during the high level in the $\theta$ band of the parietal region and in the $\alpha$ band of the fronto-temporal region. The $\frac{\beta }{(\theta +\alpha )}$ ratio index was most dominant during the medium level and lowest during the low presence level, particularly in the mid-frontal and right parietal regions.

### Classification results

The purpose of the classification was to detect presence in VR using psychophysiological features and to determine which model was the best for classifying presence within a three-class classification problem, using a One-vs-Rest approach. The classification results in Table 1 illustrates the mean performance across all 22 folds, including standard deviations for each performance metric. Results demonstrate that the MLP model produced the best performance with an average accuracy of $0.93\pm 0.03$. F1-scores for Low-vs-Rest, Medium-vs-Rest and High-vs-Rest were $0.94\pm 0.03$, $0.95\pm 0.03$ and $0.94\pm 0.03$, respectively, whilst AUC scores were $0.9 \pm 0.04$, $0.92\pm 0.04$, $0.92\pm 0.04$, respectively. The tuned hyper-parameters of the best fold of the MLP model were the logistic activation function, 1 hidden layer with 25 neurons, learning rate of 0.0001, and the Limited-memory Broyden-Fletcher-Goldfarb-Shanno (LBFGS) solver^88^.

**Table 1:** Average performance of the machine learning models.

Models	Average accuracy	Low vs rest F1 score	Medium vs rest F1 score	High vs rest F1 score	Low vs rest AUC	Medium vs rest AUC	High vs rest AUC
**MLP**	**0.93**± **0.03**	**0.94**± **0.03**	**0.95**± **0.03**	**0.94**± **0.03**	**0.93**± **0.04**	**0.92**± **0.04**	**0.92**± **0.04**
XG Boost	0.91± 0.06	0.90± 0.03	0.88± 0.04	0.90± 0.04	0.92± 0.02	0.90± 0.06	0.92± 0.01
KNN	0.83± 0.03	0.88± 0.02	0.81± 0.03	0.82± 0.02	0.86± 0.13	0.81± 0.02	0.85± 0.05
Random forest	0.83± 0.05	0.86± 0.02	0.86± 0.04	0.86± 0.15	0.86± 0.01	0.80± 0.05	0.85± 0.04
Logistic regression	0.82± 0.06	0.85± 0.02	0.84± 0.01	0.86± 0.02	0.85± 0.05	0.84± 0.04	0.85± 0.04
SVM	0.82± 0.08	0.83± 0.02	0.79± 0.02	0.85± 0.03	0.85± 0.02	0.84± 0.06	0.84± 0.02

Best performing results are in bold.

**Fig. 6:**
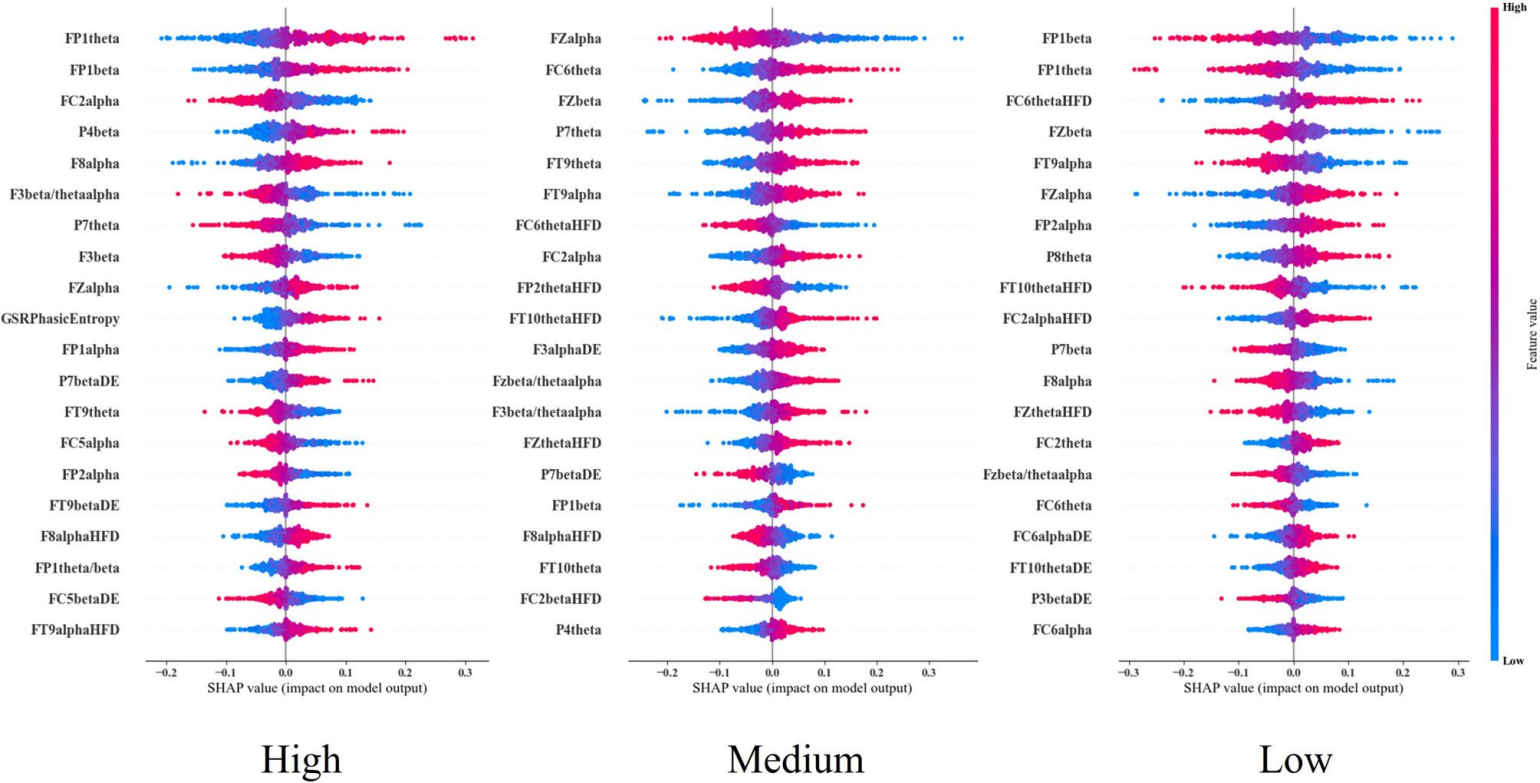
SHAP analysis results of the MLP model for the three separate classes.

Using the best performing MLP results, post-hoc analysis has been undertaken using the formula in equation (2) to calculate the Shapley values for each feature, which were used to explain the model’s prediction for that input. Due to the large number of features, Fig. 6 illustrates summary values of the top twenty most significant features across all three levels, which demonstrates alignment of each feature towards each class. When interpreting these figures, the red colony of values represents higher SHAP values, whilst the blue colony is for lower values. The y axis depicts the class values, where the positive values direct towards the concerned presence class against the other two classes.

Results illustrate a prominent pattern, with the most important features including relative $\theta$ power of FP1 for both high and low, P7, FT9 for both high and medium, FT10, P4 for medium, and P8, FC2 and FC6 for low class, relative $\alpha$ power of FC2 for both high and medium, F8 for both high and low, Fz for all the cases, FP1, FC5 for high, FP2 for high and low, FT9 for medium and low, FC2 for medium, and F8, FC6 for low class, relative $\beta$ power of FP1 for all the class, P4, F3 for high, Fz for both medium and low, P7 for low, $\frac{\beta }{(\theta + \alpha )}$ of F3 for both high and medium, Fz for both low and medium, $\frac{\theta }{\alpha }$ of FP1 for high vs all, beta band DE of P7 for both high and medium, FC5 for high, P3 for low class, theta band DE of FT10, alpha band DE of of F3 for medium, FC6 for low, theta band DE of FT10 for only low class, theta band HFD of FC6, FT10, Fz for both medium and low, FP2 for medium, alpha band HFD of FT9 for high, FC2 for medium, and beta band HFD of FC2. Finally, EDA phasic entropy played a vital role in classifying high presence.

**Fig. 7:**
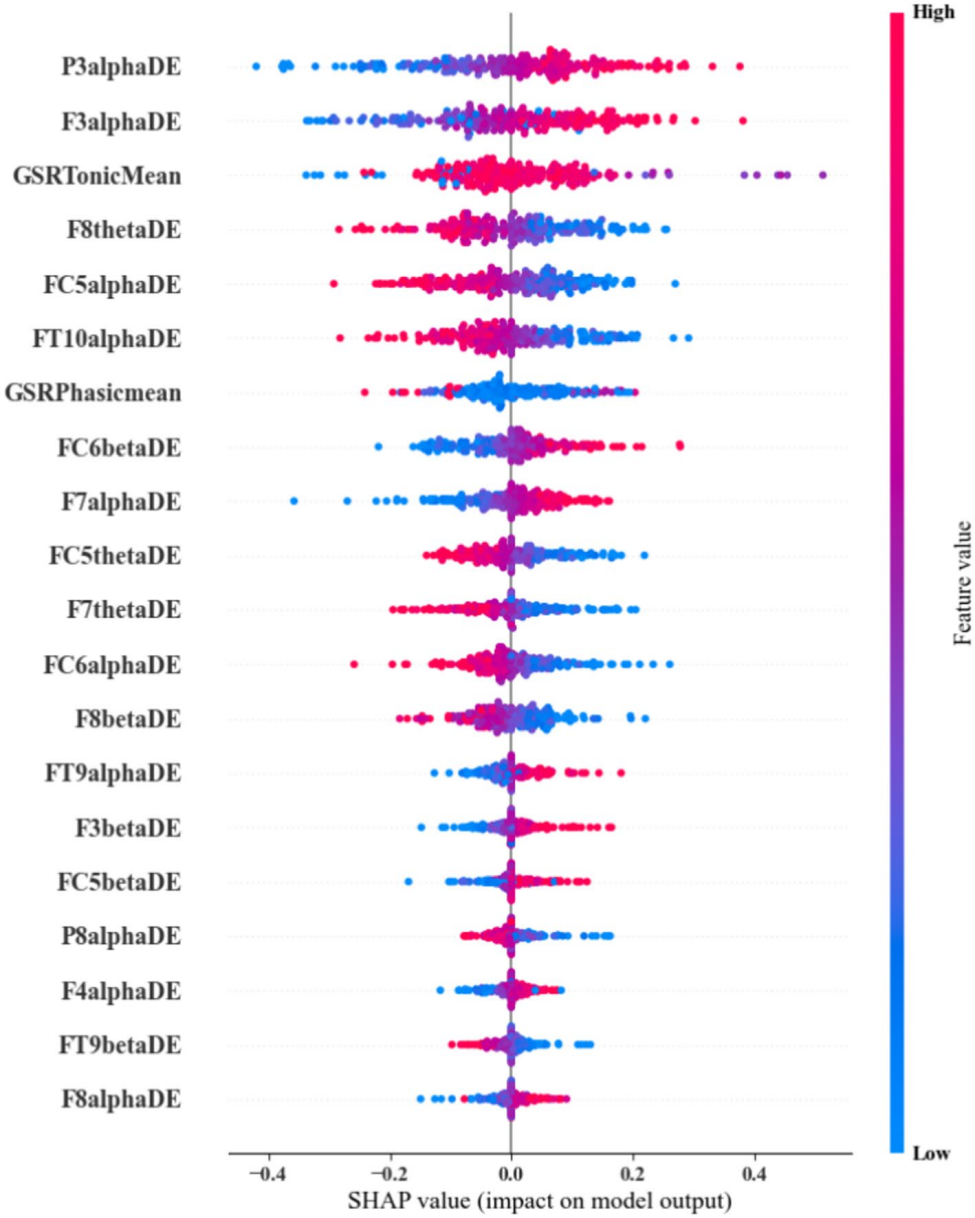
SHAP analysis results of the MLP model for the binary classification of low vs medium presence.

Furthermore, additional analysis was undertaken to distinguish significant features and model accuracy in differentiating between low and medium presence, as these levels did not include any substantial movement. Binary classification was undertaken using the low and medium classes of the same feature set. Upon application of the feature selection method, 102 features were found to be important. The MLP model was again used to classify and an average accuracy of 0.93±0.03 was obtained. An AUC of 0.95±0.03 and F1 of 0.91±0.04 were also achieved. Post-hoc SHAP results (see Fig. 7) illustrate both EDA phasic and tonic mean power significantly contributed to the classification. Interestingly, differential entropy was the most prominent feature for the EEG signals.

## Discussion

Results of this study indicate that the psychophysiological signals can be used to detect presence in VR. This has been demonstrated using three different VR environments, whereby four parameters, including visual quality, latency, audio cues, and embodiment with haptic feedback, were manipulated to alter the level of presence. This selection of parameters was based on the literature. A significant difference was found between the self-reported IPQ scores of the three levels, which validates hypothesis H1. The self-reported results also support that the environments did induce the anticipated levels of high, medium and low presence, which validates hypothesis H2. The variation in participant’s psychophysiological responses in different environments was also investigated. The findings from the ANOVA results demonstrated that the responses from both the frontal and parietal lobes were significantly different between the two levels of high and low. Our findings are also consistent with previous research^89^ that also reported changes in both frontal and parietal lobes in response to VR experiences.

Use of a genetic model for feature selection resulted in a significant reduction of the feature space by 52.4–54.6%, which is important for classification as this enables the model to learn from only the most relevant data. The derived feature set contained 82, 83, 84, 86, and 83 features for SVM, XGBoost, MLP, RF, and KNN, respectively. LOOCV was used for model validation, which is considered a robust and unbiased approach to minimise overfitting^90^. The obtained results significantly contribute to the literature, as there is limited research on utilising psychophysiological signals to detect presence. The closest similar work in this area detected presence using machine learning and data obtained from self-reports, which achieved a maximum F1 score of $80\pm 0.02\%$^91^. Crucially, our results represent a considerable improvement, with a macro average accuracy of 93% using the MLP model, with 1 hidden layer of 25 neurons and a logistic activation function. As such, these results validate hypothesis H3 to successfully classify different presence levels using psychophysiological signals.

Findings from the Shapley analysis (Fig. 6) demonstrated the features that contributed the most to each level of presence. In the case of the high presence level, relative $\beta$ and $\theta$ band power in the pre-frontal region is associated with higher presence. Additionally, the frontal region also exhibited more $\theta$ activity, which reflects better engagement^92^. Increased $\beta$ band power, beta band DE and larger relative $\beta$ band in channel P7 was also observed in the Parietal region that was associated with high presence, whilst P7 relative $\theta$ band power and relative alpha power of Fz decreased, which can also be seen in Fig. 5. Furthermore, HFD of the alpha band in the fronto-temporal region and the index $\frac{\theta }{\beta }$ were also associated with high presence. Additionally, physiological features, including tonic SCL mean, as well as both entropy and kurtosis of the phasic SCR data were also associated with high presence. This may be because a high sense of presence in the VR environment may result in increased arousal, which in turn can lead to greater variability in physiological responses^93^. As such, phasic entropy has a higher degree of contribution in classifying high levels of presence.

Additionally, in the case of the medium level, low mid-frontal relative $\alpha$ band power, low HFD of the $\theta$ band in FC6 and high relative $\beta$ band power are associated with medium presence. Furthermore, the index feature of $\frac{\beta }{\theta +\alpha }$ from both the frontal channels Fz and F3 were increased, which was also associated with medium presence. Surprisingly, medium-presence detection was aided more by lower concentrations of $\alpha$ HFD and $\theta$ HFD, respectively from F8, FP2, and FT10. HFD is a crucial component for real-time emotion detection^94^. Hence, the lower values of HFD in both the $\alpha$ and $\theta$ band, of the frontal and fronto-temporal regions were associated with the medium presence level.

Furthermore, in the case of the low presence level, the inverse trend to that of the high presence level was observed, as the relative $\theta$ band power of FP1 channel decreased in the lower presence level. Low values of $\frac{\beta }{\theta +\alpha }$ index in channel Fz were also observed in the low presence scenario. Interestingly, lower $\frac{\beta }{\theta +\alpha }$ index was associated with both the high and low presence scenarios in the F3 and Fz regions, whereas higher values in these regions were associated with medium presence. Furthermore, HFD in the fronto-central region (FC6, FC2) was significant in the low presence level, where both the $\beta$ and $\alpha$ band showed higher amplitudes. Moreover, increased P8 theta power in the Parietal region and relative alpha power of Fz were also associated with low presence.

The methods that have been discussed were applied to a three-class classification problem that considered all presence scenarios. The high presence scenario involved voluntary interaction with the environment, which played a vital role in providing a higher level of presence due to its illusory interaction^95^. However, there might be instances when the level of presence may vary without interaction, which will also affect the psychophysiological signals. This issue has been investigated by deploying a binary MLP model to classify low and medium presence. An interesting result from this analysis was that the DE feature was the most prominent feature for EEG, even when minimal to no interaction was involved. DE features are the most suitable for detecting emotion in VEs^96^ for EEG data. The SHAP analysis (Fig. 7) considered medium presence as the positive class, which was illustrated in the frontal and parietal regions with higher $\alpha$ band DE that positively contributed to the detection of the medium class. However, in the region between the frontal and temporal and frontal and central (FC6, FT10) regions, the opposite trend has been observed. Similar characteristics were illustrated in the case of the $\theta$ band, where the lower DE value was observed in the medium presence. Mixed patterns were observed in the case of the $\beta$ band, while for F8, FT9 lower values, and for FC6, F3, higher values were more prominent for the medium class. Additionally, both the tonic and phasic mean of the EDA signal were considered as important features, where higher tonic values were observed to have a positive effect, whilst higher phasic values had a negative effect on correctly classifying the data.

Our study is important as it defines the most important psychophysiological features for quantifying presence and the relationship between them, which can be seen in Fig. 6 and Fig. 7. This optimal set of features can be used in future systems to detect varying levels of presence in real time. The study has been carefully designed to focus on the regions of the brain that correspond to processing perception and engagement. This also includes the Parietal region, which controls touch and other sensory information, which leads to perception and control towards the environment^97^. This is important as exhibited neural differences between the three levels (classes) could be influenced by differences in the perception of different physical stimulation (such as sounds, visuals, etc.). As such, the brain regions that typically respond to these types of stimuli were excluded, including the parieto-temporal region, which responds to audio stimuli^98^, the central sensorimotor region for motor functions^99^, and the occipital region for processing visual stimuli^100^.

## Limitations

Whilst the results of the study are important in classifying presence, the study was not without limitations. For instance, the individual effects that each parameter had on presence were not explored. This would be beneficial to investigate in future work as this type of classification model could be used to assist in collectively determining the overall presence level produced. This would also aid in understanding the degree of adjustment required for each parameter in working towards developing an adaptive VR system. Additionally, a neutral environment was used in the study. This was a deliberate choice so that presence could be detected in the absence of a task or too much stimulation. Nevertheless, further exploration is required to understand how the study’s selected features perform in other types of environments that include specific emotional stimulation and cognitive demands.

## Conclusions and future work

The goal of the study was to detect the presence using machine learning and psychophysiological data. Using such signals, instead of solely relying on conventional self-reporting methods, allowed for a more objective measure of presence to be quantified. As there are no other studies on the classification of presence levels using these methods, the outcomes obtained from this study are, therefore, extremely important. Participants’ self-report results validated that the designed levels were significantly different in terms of the levels of presence that were experienced. The comparison of the effect of the different parameters on individuals that were used to differentiate presence was outside the scope of this paper and will be covered in subsequent work. Based on the features obtained that were of substantial importance, these will be used in future work to refine the model. This can then be used as a guide to building a framework to detect presence in real time. Future work also aims to use this real-time detection framework to develop adaptive VR environments.

## Data Availability

Owing to ethical concerns and to preserve individuals’ privacy, the raw data and code underlying this publication are not publicly accessible but may be available on request by emailing the corresponding author.
